# Transcriptional Analysis of Hair Follicle-Derived Keratinocytes from Donors with Atopic Dermatitis Reveals Enhanced Induction of *IL32* Gene by IFN-γ

**DOI:** 10.3390/ijms14023215

**Published:** 2013-02-05

**Authors:** Yoshie Yoshikawa, Yusuke Sasahara, Katsuyuki Takeuchi, Yoshimasa Tsujimoto, Takashi Hashida-Okado, Yukio Kitano, Tomoko Hashimoto-Tamaoki

**Affiliations:** 1 Department of Genetics, Hyogo College of Medicine, 1-1 Mukogawa-cho, Nishinomiya, Hyogo 663-8501, Japan; E-Mails: yoshiey@hyo-med.ac.jp (Y.Y.); yusuke-sasahara@leto.eonet.ne.jp (Y.S.); takeuchi.k@r4.dion.ne.jp (K.T.); tomokots@hyo-med.ac.jp (T.H.-T.); 2 Takara Bio Inc., Seta 3-4-1, Otsu, Shiga 520-2193, Japan; E-Mails: tsujimotoy@takara-bio.co.jp (Y.T.); okadot@takara-bio.co.jp (T.H.-O.); 3 Hyogo College of Medicine, Hyogo 663-8501, Japan; E-Mail: yukiok@m4.kcn.ne.jp

**Keywords:** gene profiling, hair follicle-derived keratinocytes, atopic dermatitis, IL32

## Abstract

We cultured human hair follicle-derived keratinocytes (FDKs) from plucked hairs. To gain insight into gene expression signatures that can distinguish atopic dermatitis from non-atopic controls without skin biopsies, we undertook a comparative study of gene expression in FDKs from adult donors with atopic dermatitis and non-atopic donors. FDK primary cultures (atopic dermatitis, *n* = 11; non-atopic controls, *n* = 7) before and after interferon gamma (IFN-γ) treatment were used for microarray analysis and quantitative RT-PCR. Comparison of FDKs from atopic and non-atopic donors indicated that the former showed activated pathways with innate immunity and decreased pathways of cell growth, as indicated by increased *NLRP2* expression and decreased *DKK1* expression, respectively. Treatment with IFN-γ induced the enhanced expression of *IL32*, *IL1B*, *IL8*, and *CXCL1* in the cells from atopic donors compared to that in cells from non-atopic donors at 24 h after treatment. *IL1B* expression in FDKs after IFN-γ treatment correlated with *IL32* expression. We hypothesized that overexpression of IL32 in hair follicle keratinocytes of patients with atopic dermatitis would lead to the excessive production of pro-IL1β and that the activation of IL1β from pro-IL1β by inflammasome complex, in which NLRP2 protein might be involved, would be augmented. This is the first report to show enhanced induction of cytokine/chemokine genes by IFN-γ in atopic dermatitis using cultured FDKs.

## 1. Introduction

The epidermis renews constantly throughout an individual’s lifetime. There are two stem cell repositories within the epidermis: the interfollicular epidermis and the hair follicle. Hair follicle stem cells can generate hair follicles, sebaceous glands, and interfollicular epidermis [1–4]. During wound-healing, keratinocyte migration enables rapid wound closure and re-epithelialization of injured skin to regenerate the body’s barrier. Hair follicle stem cells do not normally contribute to the homeostasis of the epidermis but do contribute to wound-healing following full-thickness wounds [5,6]. Cells from hair follicles are recruited into the epidermis and migrate to the wound region, but over several weeks, most of the cells from the hair follicles are eliminated from the epidermis. In this respect, hair follicle stem cells differ from interfollicular stem cells.

Hair follicle stem cells have recently attracted attention as a source of regenerative medicine. We have developed an efficient and simple method without the need for feeder cells or serum to establish human keratinocyte strains from plucked hair follicles obtained from adult scalps [7]. The cells cultured from hair follicles had been termed bulge-derived keratinocytes (BDKs). Recent studies demonstrated that more keratinocyte progenitors and stem cells exist outside the bulge region in the hair follicle in the junctional zone above the bulge and in close proximity to the sebaceous gland (isthmus) and the neck of the hair follicle (infundibulum) [8,9]. Because there were no conclusions about the origins of primary outgrowth cells from hair follicles, BDKs have been renamed follicle-derived keratinocytes (FDKs) from this article. FDKs are more refractory to differentiation induction by CaCl_2_, as indicated by their lower expression of *KRT10* and higher expression of *ITGA6* than neonatal foreskin-derived epidermal keratinocytes (NHEKs) [7].

Patient-derived FDKs, individually established without invasive biopsies, may be an ideal cell source to study skin diseases *in vitro*. We previously reported the use of FDKs in *in vitro* sensitizing tests and that these cells might be a powerful tool to evaluate donor-to-donor variation in response to sensitizers [10]. Furthermore, we established FDKs from adult donors with atopic dermatitis (AD) or anamnesis of AD and non-atopic donors: AD-FDKs and Non-AD-FDKs, respectively. Atopic diseases are characterized by IgE sensitization to environmental allergens. Intensive research on T helper (Th) 2 cell responses is continuing to elucidate the mechanisms involved in the development of AD [11]. In addition, skin barrier dysfunction promotes the development and severity of AD [12]. Loss-of-function mutations in the filaggrin (*FLG*) gene have been shown to be strongly linked to the phenotype of AD [13,14]. Although mutations in *FLG* could serve as predisposing factors for AD and be generally associated with more severe AD, these mutations do not necessarily lead to AD [15].

Recent reports have revealed that keratinocytes are highly immunoreactive cells that exert substantial control over the acute and chronic phases of skin inflammation by means of cytokine/chemokine production and surface molecule expression [16,17]. Keratinocyte apoptosis is an important mechanism of eczema and spongiosis in patients with AD and is mediated by IFN-γ, which is secreted by Th1 cells [18]. Rebane *et al*. reported a comparative study of IFN-γ-induced apoptosis using skin keratinocytes isolated from patients with AD and healthy donors [19]. They found that keratinocytes of patients with AD exhibit increased IFN-γ-induced apoptosis compared to keratinocytes from healthy subjects. They also reported that some genes, including *CCDC109B* and *IFI35*, were overexpressed in AD skin lesions and were induced by IFN-γ in primary keratinocytes. These data demonstrate that increased IFN-γ responses in the keratinocytes of patients with AD might contribute to the development of AD. Furthermore, interleukin 32 (IL32), a proinflammatory cytokine produced by keratinocytes, is reported to modulate the apoptosis of these cells and contribute to the pathophysiology of AD [20].

The end goal of our study is to distinguish atopic eczema from non-atopic eczema. The aim of the present study was to gain insight into gene expression signatures that can distinguish AD from non-AD without skin biopsies and predict the pathogenesis of AD. We conducted a comparative study of gene expression in FDKs. This study provides knowledge about the dysfunctions of the follicular keratinocyte-based skin defense system in AD patients but not about the interactions with immune effectors and Langerhans cells taking place in the skin. It was reported that NHEKs, which are easy-to-use epidermal keratinocytes, have a gene expression profile that is similar to that of skin biopsies, except for the genes associated with terminal differentiation [21].

## 2. Results and Discussion

### 2.1. Gene Expression Profile of FDKs

To survey the characteristics of gene expression in FDKs, we compared the gene expression profiles of these hair follicle-derived cells with those of epidermal cells (NHEKs) in culture conditions. Cells were harvested during log-phase growth, and their gene expression profiles were examined using microarrays. The gene expression profile of FDKs was similar to that of NHEKs. Both FDKs and NHEKs showed abundant expression of genes encoding structural components (cytokeratins and small proline-rich proteins), molecules associated with cell-cell and cell-extracellular matrix adhesion (integrins and desmosomal proteins), and proteins involved in cornification of the skin (S100 calcium-binding proteins, annexins, and cystatins) (Table S1). The genes showing differential expression between FDKs and NHEKs are described in Table S2. The top three RefSeq genes that varied significantly in their expression levels between FDKs and NHEKs were *DKK1*, *PLOD2*, and *CXCL1* (Table S2).

We confirmed the differential expression of these genes by real-time RT-PCR (data not shown). The expression of *DKK1*, which encodes an inhibitor of the Wnt signaling pathway, was lower in FDKs than in NHEKs, while the expression of other Wnt-related genes, such as *WNT5B*, *FZD8*, and *GSK3B*, was higher (Table S2). Expression of *PLOD2*, encoding an enzyme that catalyzes the post-translational modification of collagen, was higher in FDKs than in NHEKs, as was the expression of the *P4HA2* and *LOXL2* genes that encode similar collagen-modifying enzymes (Table S2). Several collagen-encoding genes were expressed at higher levels in the former than in the latter: *COL4A2*, *COL5A1*, *COL5A2*, *COL6A1*, *COL6A2*, *COL7A1*, *COL8A2*, and *COL18A1* (Table S2). Expression of *CXCL1*, encoding melanoma growth stimulating activity-alpha, which promotes neutrophil infiltration, was higher in FDKs than in NHEKs, as was the expression of the other chemokine genes *CXCL2*, *CXCL3*, *CXCL14*, and *CX3CL1* (Table S2). Pathway analysis of the gene set showing differential expression between FDKs and NHEKs indicated that migration, adhesion, cell spreading, and the MAP kinases cascade were enhanced in FDKs compared to that in NHEKs (Tables 1 and S3). These gene expression characteristics suggested that FDKs are more active in wound-healing and innate immunity than NHEKs. These results indicated that FDKs might be an optimal tool to evaluate the innate immune response to stimulants or stressors.

### 2.2. Genes Differentially Expressed between AD-FDKs and Non-AD-FDKs

We compared the gene expression profiles of 11 AD-FDKs with 7 Non-AD-FDKs. For the constitutive molecules acting as a skin barrier, the expression of most genes coding for cytokeratins, desmosomal proteins, integrin α3β1, fibronectin 1, small proline-rich proteins, and calcium binding proteins was not significantly different between AD-FDKs and Non-AD-FDKs (Table S4). No significant difference in *FLG* gene expression between AD-FDKs and Non-AD-FDKs was detected by real-time RT-PCR (data not shown). However, the expression levels of *LAMB3* and *CD81* were higher in AD-FDKs than in Non-AD-FDKs (Table S4). The *LAMB3* gene encodes the beta 3 chain of laminin 5, whose synthesis in cultured human keratinocytes is stimulated by acute wound fluid [22]. The *CD81* gene encodes a member of the tetraspanin family that is involved in keratinocyte migration during wound-healing [23]. Enhanced expression of CD81 in epidermal dendritic cells from AD was reported [24], but this is the first report concerning one of the *LAMB3* gene in AD.

**Figure 1:**
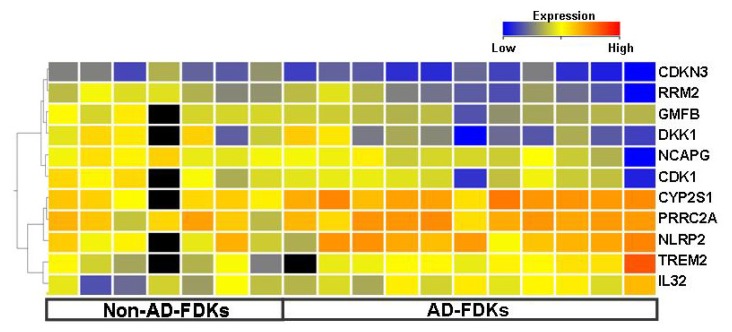
Genes showing differential expression between FDKs from donors with atopic dermatitis (AD) and from non-atopic controls (Non-AD) by microarray analysis, as determined by comparison analysis using a permutation test. The log_2_ ratio (the Cy5 to Cy3 fluorescence intensity using the same Cy3-labelled probe, consisting of a mixture of NHEK extracts and Universal Human Reference RNA, as a reference) was used as a expression level. For comparative studies of gene expression between AD-FDKs and Non-AD-FDKs, a permutation test was performed (number of permutations: 500) and 827 genes were extracted. In this figure, 11 genes that exhibit a difference of at least 2-fold and meet the criterion *p* < 0.05 are visualized as the tree after hierarchical clustering (Distance; pearson uncentered, Linkage; complete).

Eleven genes exhibited a difference of more than 2-fold in expression: *DKK1*, *RRM2*, *CDKN3*, *CDK1*, *GMFB*, and *NCAPG* (decreased in AD-FDKs) and *TREM2*, *IL32*, *CYP2S1*, *PRRC2A*, and *NLRP2* (increased in AD-FDKs) (Figure 1, Table S5). Almost all of these genes are associated with the control of cell growth and/or the production of pro-inflammatory cytokines. Molecular pathways associated with cell growth were suppressed, whereas those associated with apoptosis were enhanced in AD-FDKs compared with Non-AD-FDKs (Tables 2 and S6). In addition, pathway analysis indicated that the former showed greater activation of stress-response, including oxidative-stress-response, than the latter.

**Figure 2:**
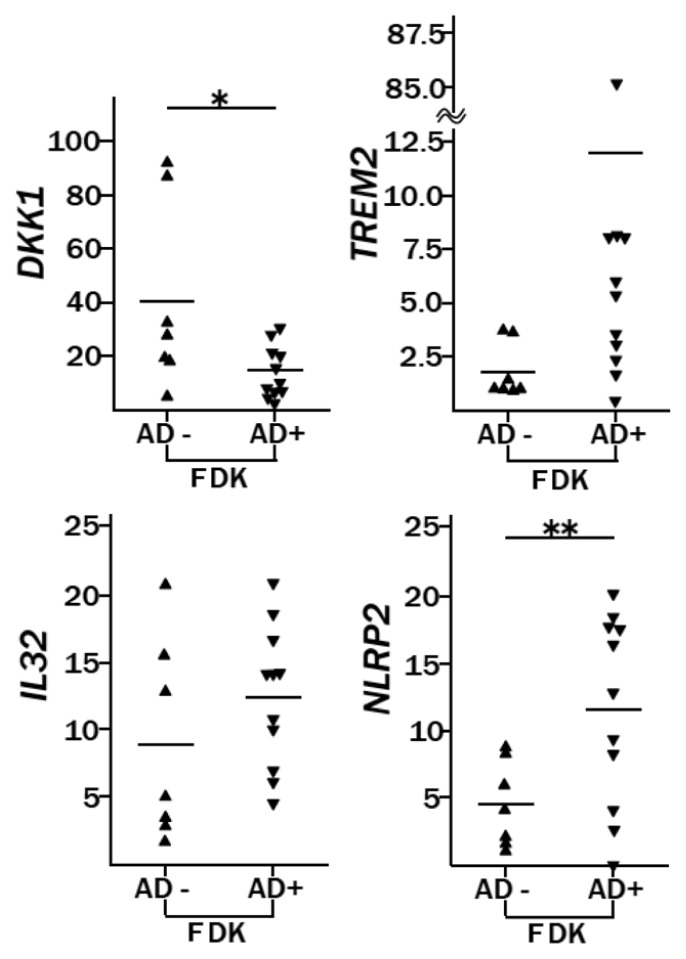
Genes showing differential expression between FDKs from donors with atopic dermatitis (AD+) and from non-atopic controls (AD−) by real-time RT-PCR analysis. The expression level of each gene analyzed by real-time RT-PCR is shown relative to that in NHEK cells (reference cells, lot no. 3C1272). The reference cell expression level was set at 1 for all but one gene (*DKK1*, for which the level was set at 100). The data are presented as the mean values of two replicate tests. ▲: Non-AD-FDKs (AD−), *n* = 7; ▼: AD-FDKs (AD+), *n* = 11; —: the mean of each group; * *p* < 0.05 and ** *p* < 0.01.

We demonstrated the significant decrease of *DKK1* in AD-FDKs by real-time RT-PCR, but highly variable expression levels of this gene were detected in the non-atopic type (Figure 2). The significance of Wnt signaling in AD is not known, but altered signaling has been reported in psoriatic skin: the WNT5a protein increases, and the WIF1 protein, the inhibitor of this signaling, decreases [25]. It has been reported that treatment of keratinocytes with DKK1 increases the proliferation and decreases the melanin uptake of these cells, and that DKK1 treatment of reconstructed skin *in vitro* induces a thicker and less pigmented epidermis [26]. In our experience, the percentage of follicles with primary outgrowth out of the total follicles inoculated in AD was lower than that in Non-AD (AD: 38.8 ± 18.7, Non-AD: 50.9 ± 23.5, *p* < 0.05), and the growth rate of AD-FDKs was slower than that of Non-AD-FDKs after passage 4 (data not shown). One factor causing the vulnerability of the skin of AD patients might be their inherent low expression of *DKK1* by keratinocytes.

Similarly, we used real-time RT-PCR to show that *TREM2*, *IL32*, and *NLRP2*, which are all associated with the production of pro-inflammatory cytokines, were highly expressed in AD-FDKs, although this trend was only statistically significant for *NLRP2* (Figure 2). Pattern recognition receptors, including Toll-like receptors (TLRs) and nucleotide-binding oligomerization domain (NOD)-leucine rich containing protein family members (NLRs) [27], recognize pathogen-associated molecular patterns, and keratinocytes express these molecules. Between AD-FDKs and Non-AD-FDKs, there were no significant differences in gene expression for the genes encoding TLRs [*TLR1*, *TLR2*, *TLR3*, and *TLR5*; somewhat higher expression in AD-FDKs for *TLR9*; and inconclusive results for *TLR4* because of low expression] (data not shown). AD-FDKs had a 2.5-fold higher expression of *NLRP2* than Non-AD-FDKs (*p* = 0.006) (Figure 2). The NLRP2 protein may be involved in protein complexes that activate proinflammatory caspases [28]. Activation of the inflammatory caspases requires the assembly of a unique intracellular complex, designated the inflammasome, that proceeds to cleave and activate IL18 and IL1β; inflammatory caspase 1 cleaves the precursor form, pro-IL1β, to the active form, IL1β [29].

Except for a few genes, most of the differentially expressing genes between AD-FDKs and Non-AD-FDKs identified in our studies were not found in previous studies using AD and Non-AD skin biopsies. The differences between our study and the previous ones using skin biopsies might be due to cell components (using keratinocytes only or whole tissue samples) and their origin (follicular or interfollicular stem cells).

### 2.3. Enhanced Induction of IL32 and IL1B by IFN-γ Treatment in AD-FDKs Compared to Non-AD-FDKs

We analyzed the responses to IFN-γ (50 ng/mL), TNF-α (50 ng/mL), Zymosan (10 μg/mL), and ultraviolet B radiation (50 mJ/cm^2^) in AD-FDKs and compared them with those of Non-AD-FDKs. The comparison analysis indicated that IFN-γ treatment resulted in the greatest number of genes showing significantly differential expression between the two groups of FDKs (data not shown). Thus, we focused on IFN-γ in this study. Because the number of viable cells apparently decreased after the 72 h IFN-γ treatment, we analyzed gene expression after 24 h. The gene expression profile after a 24 h treatment revealed pro-apoptosis activation in both FDKs and NHEKs; for example, the *UBD* and *STAT1* genes were remarkably upregulated (Figure S1).

**Figure 3:**
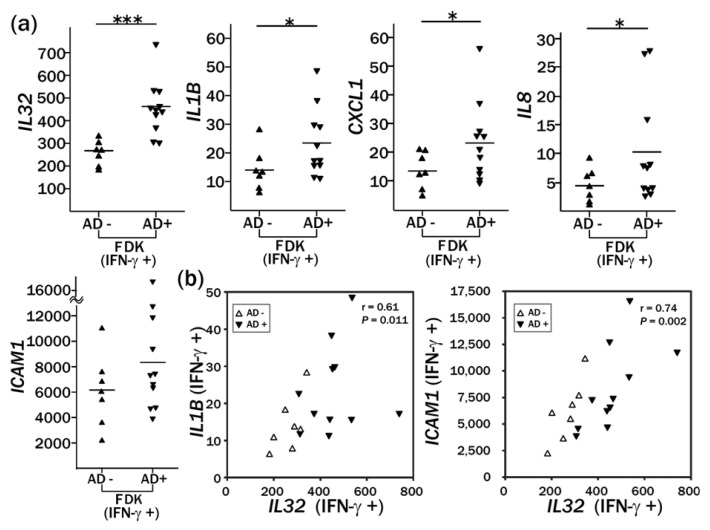
Enhanced expression of *IL32*, *IL1B*, *CXCL1*, *IL8*, and *ICAM1* after stimulation with IFN-γ in FDKs derived from donors with atopic dermatitis (AD+) compared to the expression in those from non-atopic controls (AD−) (**a**). (**b**) Correlation of gene expression between *IL32* and *IL1B* and between *IL32* and *ICAM1* in FDKs. FDK cultures were treated with IFN-γ at a concentration of 50 ng/mL and were incubated for 24 h. The expression level of each gene analyzed by real-time RT-PCR is shown relative to that in an NHEK strain without IFN-γ treatment (ID: NHEK 1, lot no. 3C1272). The reference cell expression level was set at 1 for all of the genes. ▲ or △: Non-AD-FDKs (AD−), *n* = 7; ▼: AD-FDKs (AD+), *n* = 11; —: the mean of each group; * *p* < 0.05, *** *p* < 0.001.

In a time-course study using a microarray, many genes were found to show a more rapid transcriptional response to IFN-γ in the AD-FDKs than in the Non-AD-FDKs, including *IL32*, *CCDC109B* and *IFI35* (Figure S1). Because it was reported that IL32 contributes to the pathophysiology of AD [20], we quantified the expression of *IL32* and its associated genes (*IL1B*, *CXCL1*, *IL8*, and *ICAM1*) in 18 FDKs by real-time RT-PCR. The expression of *IL32* and *ICAM1* increased at 24 h after IFN-γ treatment by 6- to 100-fold for *IL32* and 20- to 750-fold for *ICAM1* (data not shown). The induction of *IL1B*, *CXCL1*, and *IL8* by IFN-γ was less remarkable. AD-FDKs showed significantly higher expression of *IL32*, *IL1B*, *CXCL1*, and *IL8* than Non-AD-FDKs, but the upregulation of *ICAM1* was not significant: *IL32: p* = 0.0002; *IL1B: p* = 0.026; *CXCL1: p* = 0.031; *IL8: p* = 0.038; and *ICAM1: p* = 0.09 (Figure 3a). Gene expression of *IL1B* and *ICAM1* in FDKs after IFN-γ treatment correlated with that of *IL32: IL1B*: Spearman *r* = 0.61, *p* = 0.011; and *ICAM1*: Spearman *r* = 0.74, *p* = 0.002 (Figure 3b).

Our data suggest that patients with AD might possess unknown factors that promote the production of IL32. Therefore, we speculated that the promoted interaction, mediated by ICAM1, between hair follicle keratinocytes and the Th1 cells that produce IFN-γ would cause overexpression of IL32 in the skin of patients with AD. IL32 might lead to excessive production of pro-IL1β in keratinocytes, monocytes, and Langerhans cells, and the activation of IL1β from pro-IL1β by inflammasome complex, in which NLRP2 might be involved, would be augmented. This augmentation would induce a large panel of cytokines/chemokines, including IL8 and CXCL1, and other inflammatory mediators acting in a signaling cascade on target cells, as well as within autocrine loops in hair follicle keratinocytes.

## 3. Experimental Section

### 3.1. Plucked Hair

Eighteen Japanese volunteers between 26 and 77 years of age donated plucked hairs under conditions of informed consent; 11 of these volunteers had AD or anamnesis of AD diagnosed by a dermatologist or paediatrician in childhood (average age = 37.3 years; 7 males and 4 females), and seven were non-atopic controls (average age = 46.4 years; 5 males and 2 females). This study was performed according to the Declaration of Helsinki, and the procedure was approved by the ethical board of the Hyogo College of Medicine. Approximately 10 plucked hairs were obtained by removing hairs from the temporal scalp with depilation forceps. The hair samples were trimmed to remove the end grasped by the forceps and immediately dipped in Keratinocyte-SFM medium (DK-SFM; GIBCO/Invitrogen, Carlsbad, CA, USA).

### 3.2. Cell Culture and RNA Extraction

Primary cell cultures from plucked hair follicles were established as described previously [7]. NHEKs in the first passage were purchased from Kurabo (Osaka, Japan). When the FDKs and NHEKs reached 70% confluency in DK-SFM medium, they were trypsinized and transferred to a new culture plate. Eighteen FDKs from 18 donors and six NHEKs from different lots, both after three to four passages, were used for gene expression analysis. The culture medium was renewed on day 3, and cells in log-phase were harvested on day 4. FDK cultures were treated with recombinant human interferon gamma (IFN-γ; Shionogi & Co., Ltd., Osaka, Japan) at a concentration of 50 ng/mL the day after changing the medium and were incubated for 24 h.

Total RNA was isolated using TRIzol reagent (Invitrogen) and purified using an RNeasy kit (QIAGEN, Hilden, Germany) according to the manufacturer’s protocol.

### 3.3. Microarray Analysis

A custom-made cDNA microarray (16,272 human genes, Takara Bio, Shiga, Japan, GEO platform: GPL7687 and GPL7688) was used for microarray analysis. For hybridization, each total RNA was labeled with Cy3 or Cy5 monoreactive dyes (Amersham, Buckinghamshire, UK) using an RNA Transcript SureLABEL™ Core Kit (Takara Bio), according to the manufacturer’s instructions. For each sample, 4 micrograms of total RNA extracted from 18 FDKs (11 AD-FDKs and 7 Non-AD-FDKs) or 6 NHEKs from different lots was labeled with Cy5, respectively. The Cy3-labelled probes, consisting of a mixture of NHEK extracts and Universal Human Reference RNA (Stratagene Corporation, La Jolla, CA, USA) to obtain FDK-specific differential gene expression profiles that are distinct from NHEK patterns, were used as references. By two-color microarray-based gene expression analysis using the same probe as a reference for all experiments, the differences in the ratio among the microarrays indicated the differences in the amounts of RNA between the analyzed samples in spite of two-color analysis. The Cy3- and Cy5-labelled probes were mixed with Human Competitor I (Takara Bio) and suspended in 55 μL of hybridization buffer containing 50% formamide, 6× SSC, 0.2% SDS, 5× Denhardt’s, and 0.2 mg/mL denatured salmon sperm DNA. The two-color mixed probe prepared from each sample was denatured at 70 °C for 10 min and applied to each microarray. Hybridization was performed at 70 °C for 16 h, and the microarray slides were washed three times with 2× SSC, 0.2% SDS at 65 °C for 10 min and rinsed once with 0.05× SSC at room temperature. The microarray slides were then spin-dried. Microarray scanning was performed using an Affymetrix 428 Array Scanner (Affymetrix Inc., Santa Clara, CA, USA). The images were processed using the analysis software program ImaGene version 6.0 (BioDiscovery Inc., Hawthorne, CA, USA). For each hybridized spot, the background intensity was subtracted and normalized by the global LOWESS normalization method. The log_2_ ratio of Cy5 to Cy3 fluorescence intensity was used as the gene expression value. Probes with poor-quality signals (signal/noise < 1.2) and those with null data at a frequency of more than 35% in all samples were filtered out using the microarray data analysis software Expressionist (GeneData AG, Basel, Switzerland); after this procedure, 13,722 probes remained. For comparative studies of gene expression, a permutation test was applied with GeneSpring software (Agilent Technologies Inc., Santa Clara, CA, USA) to reduce the false discovery rate. Array data have been deposited in Gene Expression Omnibus (GEO): GSE13709. Pathway analysis was performed to identify the significant biological functions and transcription factors causing the differences in gene expression using Ingenuity Pathways Analysis (Ingenuity Systems, Redwood City, CA, USA). In this analysis, prediction of the activation state of each biological function was defined using a regulation *z*-score; *z*-scores greater than 2 (activating) or less than −2 (inhibiting) were considered significant.

### 3.4. Real-Time RT-PCR

Total RNA (1 μg) was reverse-transcribed using a PrimeScript^®^ RT reagent kit (Perfect Real Time) (Takara Bio Inc., Shiga, Japan). PCR was conducted in a reaction mixture containing 1× SYBR^®^*Premix Ex Taq*™ II (Takara Bio), 10 ng of cDNA, and the following primers: *CXCL1*: 5′-AGCTTGCCTCAATCCTGCATCC-3′ and 5′-TCCTTCAGGAACAGCCACCAGT-3′, *DKK1*: 5′-ACCAGACCATTGACAACTACCAGC-3′ and 5′-TAATTCCCGGGGCAGCACAT-3′, *ICAM1*: 5′-ATGCCCAGACATCTGTGTCC-3′ and 5′-GGGGTCTCTATGCCCAACAA-3′, *IL1B*: 5′-TGTACCTGTCCTGCGTGTTGAA-3′ and 5′-AGGTGCTGATGTACCAGTTGG-3′, *IL32*: 5′-AGCTCTGACCTGGTGCTGTC-3′ and 5′-ACATCACCCAGTCTCAGGCATTCT-3′, *IL8*: 5′-GAGAGCTCTGTCTGGACCCCAA-3′ and 5′-ATCTGGCAACCCTACAACAGACCCA-3′, *NLRP2*: 5′-CATTCTGCGTCAAGCACTGTCG-3′ and 5′-CCGTCCAGAAAGGAAGCATGTG-3′, *TREM2*: 5′-ATGATGCGGGTCTCTACCAGTG-3′ and 5′-GCATCCTCGAAGCTCTCAGACT-3′, *HPRT1*: 5′-GATGGTCAAGGTCGCAAGCTT-3′ and 5′-CTGGCGATGTCAATAGGACTCCAG-3′. The expression of each gene was calculated by the comparative threshold cycle method (ΔΔCt) and normalized to that of hypoxanthine phosphoribosyltransferase 1 (*HPRT1*). The expression level of each gene is shown relative to that of an NHEK strain (ID: NHEK 1, lot no. 3C1272). The data are presented as the mean values of two replicate tests.

### 3.5. Statistical Analysis

Comparative studies of gene expression between FDKs and NHEKs and between AD-FDKs and Non-AD-FDKs were performed with the Welch test; the filtering criterion was *p* < 0.05. For correlation analysis, the Spearman correlation test was used.

## 4. Conclusions

Comparison of FDKs from AD and Non-AD donors using microarray analysis indicated that the former showed activated pathways involved in innate immunity and decreased pathways of cell growth, as indicated by increased *NLRP2* expression and decreased *DKK1* expression, respectively. Independent of immune effectors, IFN-γ treatment induced significantly enhanced expression levels of *IL32*, *IL1B*, *IL8*, and *CXCL1* in the cells from AD compared to the expression levels in cells from Non-AD donors. FDKs cultured from donors with AD exhibited the features that induced overexpression of proinflammatory cytokine genes in response to IFN-γ. While we were preparing this manuscript, it was reported that hair follicle keratinocytes modulate the repopulation of Langerhans cells from their precursors and the entry of their precursors into the epidermis *via* chemokine production [30]. These data indicate that hair follicle keratinocytes might play important roles in the control of inflammation in skin diseases.

FDKs can be individually established without invasive biopsies and are likely to be widely applicable to the study of the genetic factors that influence defense and stress responses in individuals.

## Figures and Tables

**Table 1:** Pathway analysis to identify the biological functions of the genes showing differential expression profiles between follicle-derived keratinocytes (FDKs) and neonatal foreskin-derived epidermal keratinocytes (NHEKs).

Function annotation	*p* value	Predicted activation state in FDKs	Regulation z-score
Migration of cells	6.58 × 10^−7^	Increased	3.025
Cell movement	1.77 × 10^−6^	Increased	2.942
Adhesion of immune cells	8.47 × 10^−4^	Increased	2.893
Cell spreading	5.16 × 10^−5^	Increased	2.857
Transmigration of cells	4.98 × 10^−6^	Increased	2.635
Binding of cells	1.93 × 10^−3^	Increased	2.438
MAPKKK cascade	1.80 × 10^−3^	Increased	2.387
Expression of RNA	1.88 × 10^−3^	Increased	2.298
Autophagy	1.41 × 10^−4^	Increased	2.26
Regression of embryonic tissue	2.10 × 10^−3^	Increased	2.2
Initiation of cell death	2.04 × 10^−3^	Increased	2
Alignment of chromosomes	1.60 × 10^−6^	Decreased	−2.608

Pathway analysis of the microarray data sets using Ingenuity Pathways Analysis was performed for the genes showing differential expression between FDKs and NHEKs. The *p* values for each function, indicating their significance, are shown. Prediction of the activation state of each biological function was defined using a regulation z-score; z-scores greater than 2 (activating in FDKs) or smaller than −2 (inhibiting in FDKs) were considered significant.

**Table 2:** Pathway analysis of the genes showing differential expression between FDKs derived from donors with atopic dermatitis and from non-atopic controls.

Function annotation	*p* value	Predicted activation state	Regulation *z*-score
Cellular response to therapeutics: sensitivity of cells	0.0335	Increased	2.543
Molecular transport: concentration of glutathione	0.0126	Increased	2.416
Cell death	0.0001	Increased	2.409
Apoptosis	0.0078	Increased	2.048
Cell proliferation	0.0058	Decreased	−3.071

Prediction of the activation state of each biological function in FDKs derived from donors with atopic dermatitis was determined using a regulation z-score.

## Supplementary Files

Supplementary File 1 Supplementary Information (PDF, 319 KB)

Supplementary File 2 Supplementary Table (XLS, 283 KB)
