# Supplementary material for: Transcriptional Analysis of Hair Follicle-Derived Keratinocytes from Donors with Atopic Dermatitis Reveals Enhanced Induction of IL32 Gene by IFN-γ
Source: Int J Mol Sci. 2013 Feb 5;14(2):3215–27. doi: 10.3390/ijms14023215 (PMC3588040; doi:10.3390/ijms14023215)

Supplementary Information

Figure S1. Transcriptional responses of FDKs and NHEKs to IFN-γ. FDKs derived from AD and from Non-AD, and two different lots of NHEKs were treated with IFN-γ (50 ng/mL) and subjected to time-course analysis: 0, 6, 12, 24 h. The expression of each gene of 152 genes showing periodic patterns in response to IFN-γ was visualized as the tree after hierarchical clustering (Distance; pearson uncentered, Linkage; complete).

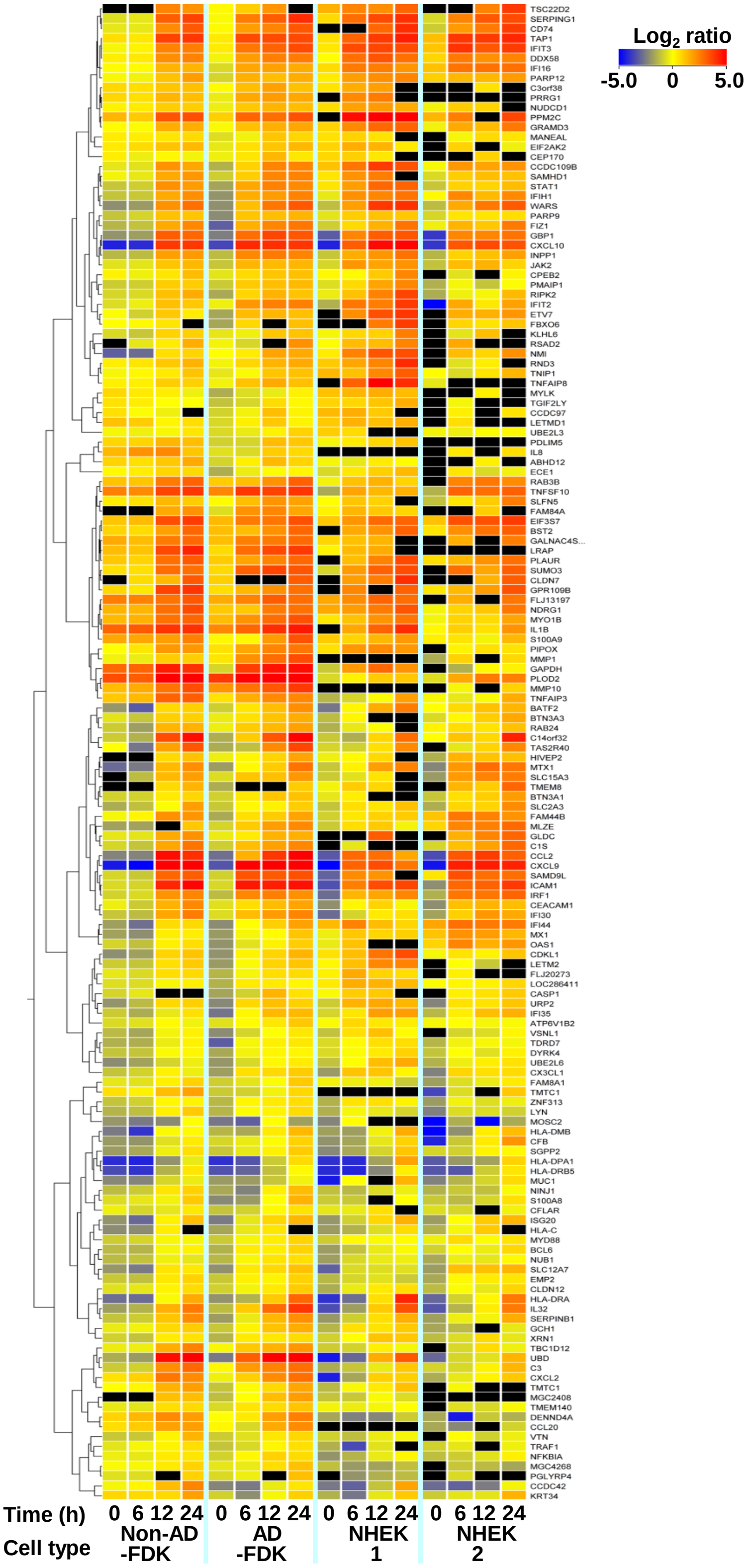

Supplement: Supplementary File 1 — Supplementary Information (PDF, 319 KB) [file ijms-14-03215-s001.pdf]
